# A swirl ERCP during combined endoscopic-radiological-surgical management of a late-onset post-traumatic obstructive jaundice

**DOI:** 10.1055/a-2302-7617

**Published:** 2024-05-29

**Authors:** Giacomo Emanuele Maria Rizzo, Lucio Carrozza, Salvatore Gruttadauria, Alessandro Bertani, Roberto Miraglia, Ilaria Tarantino, Mario Traina

**Affiliations:** 1 Endoscopy Service, Department of Diagnostic and Therapeutic Services, IRCCS-ISMETT, University of Pittsburgh Medical Center Italy (UPMC Italy), Palermo, Italy; 2 Department of Surgical, Oncological and Oral Sciences, University of Palermo, Palermo, Italy; 3 Department of the Treatment and Study of Abdominal Diseases and Abdominal Transplantation, IRCCS-ISMETT, University of Pittsburgh Medical Center Italy (UPMC Italy), Palermo, Italy; 4 Department of General Surgery and Medical-Surgical Specialties, University of Catania, Catania, Italy; 5 Unit of Thoracic Surgery and Lung Transplantation, Department for the Treatment and Study of Cardiothoracic Diseases and Cardiothoracic Transplantation, IRCCS-ISMETT, University of Pittsburgh Medical Center Italy (UPMC Italy), Palermo, Italy

A 47-year-old man suffered diaphragmatic laceration in a road trauma around the age of 30, with associated thoracic herniation of the right colon and part of the duodenum, atelectasis of the right lower lung lobe with median dislocation of the liver, atrophy of the right liver lobe, and compensatory hypertrophy of the left liver lobe. After 15 years, he developed an episode of cholestasis and jaundice, and was admitted to another center for initial evaluation, involving viral and serological screening for liver causes of jaundice, including the reported intake of ibuprofen. All evaluations were negative, so abdominal contrast-enhanced computed tomography (CT) scan was performed and showed dilation of the common bile duct (CBD) and intrahepatic biliary ducts (IBDs), without evidence of lithiasis and/or pathological interruptions. Liver biopsy was performed and histological evaluation was suspicious for “vanishing bile duct syndrome,” so oral steroid therapy was started, but had no clinical benefit.

**Fig. 1:**
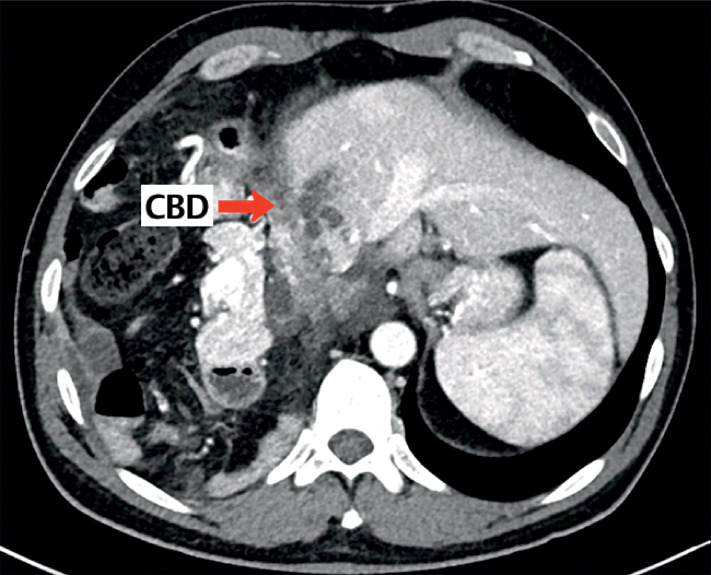
Angled bile duct.

He was referred to our tertiary center, where a further chest-abdomen CT scan showed the well-known voluminous right transdiaphragmatic hernia, with CBD displaced in the thorax, creating a 90-degree angle with consequent mechanical stenosis ( Fig. 1 ). Following multidisciplinary evaluation, it was decided to perform surgery to repair the diaphragmatic hernia, cholecystectomy, and partial hepatectomy (VI and VII segments).

**Fig. 2:**
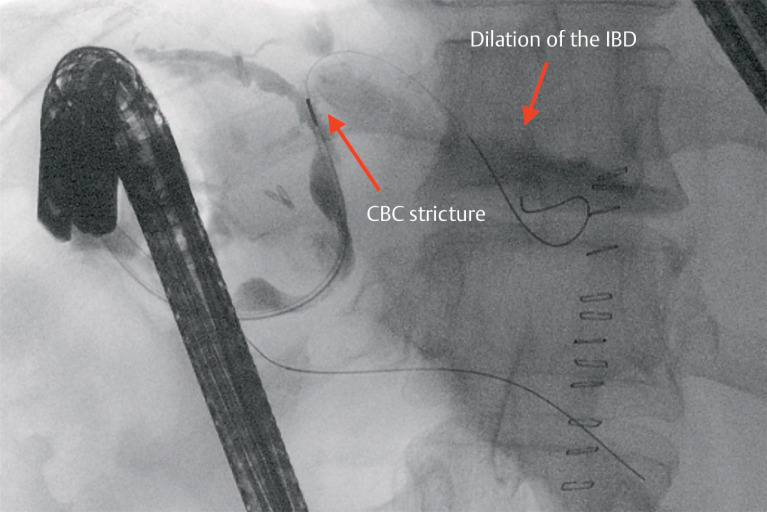
Common bile duct stricture and dilation of the intrahepatic biliary duct.

**Fig. 3:**
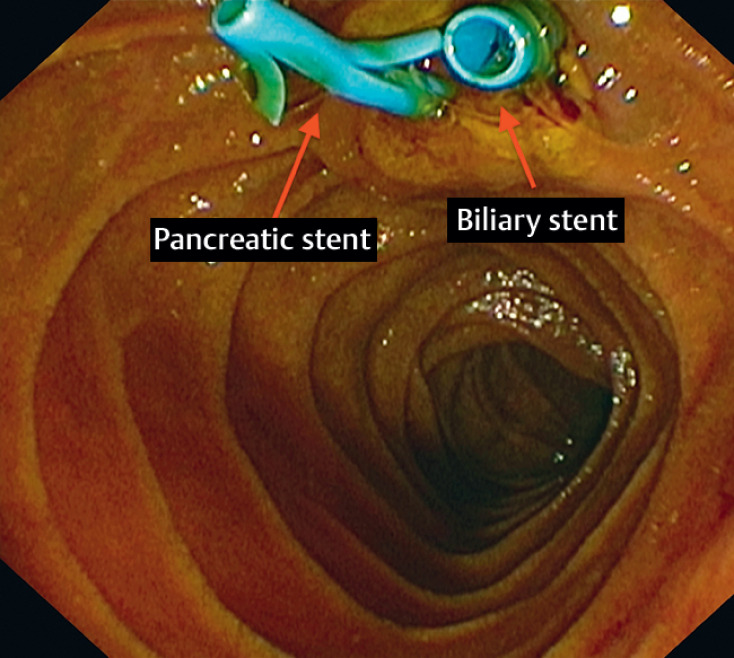
Biliary and pancreatic plastic stents.

Intraoperative cholangiography was performed from the cystic duct remnant that showed normalization of the straightness of the main bile duct in the absence of biliary leakage, with dilation of the IBD upstream of an angle of the common hepatic duct. Despite undergoing surgery, the patient underwent subsequent endoscopic retrograde cholangiopancreatography (ERCP) because of persistent jaundice, which was challenging due to the novel anatomical arrangement ( Video 1 ). Cholangiography showed an angled CBD at the hilar confluence, causing stricture of the common hepatic duct and consequent dilation of the IBD, predominantly on the left ( Fig. 2 ). Sphincterotomy was performed, followed by placement of both biliary and pancreatic plastic stents ( Fig. 3 ).

Multidisciplinary management of a rare case of obstructive jaundice.Video 1

**Fig. 4:**
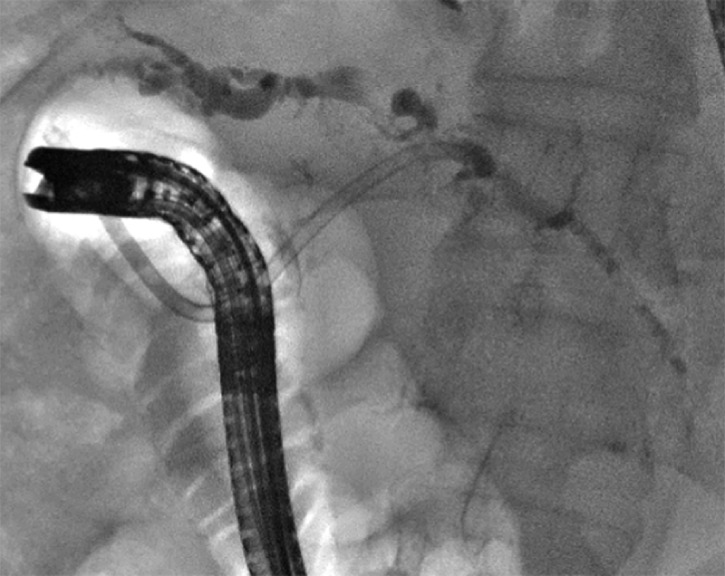
Placement of a biliary plastic stent with an endoscopic-radiologist rendezvous.

**Fig. 5:**
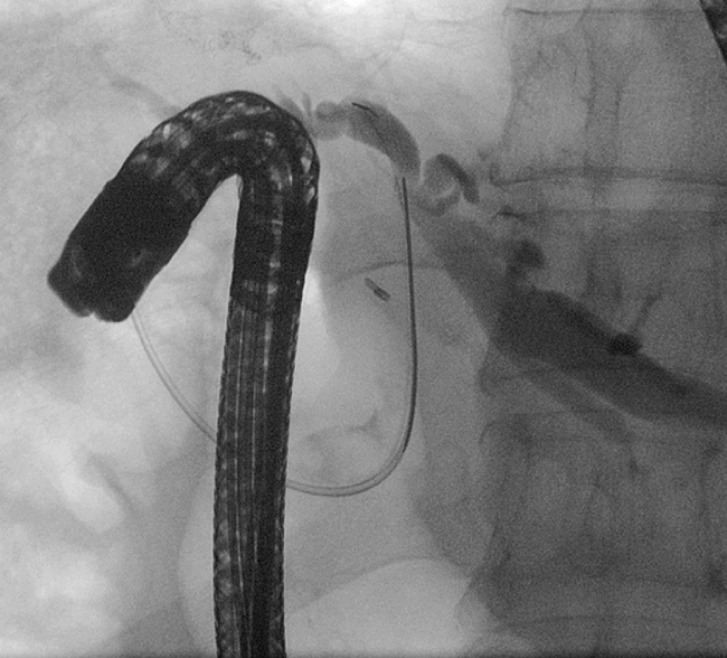
Cholangiogram without leakage.

Approximately 20 days after surgery, the chest drainage showed biliary contents, and the patient developed fever and cough. Percutaneous cholangiography showed extravasation of contrast dye from the hepatic duct at the hilum, so an external–internal biliary catheter was placed and subsequently replaced by an internal plastic stent (12 cm × 10 Fr) with an endoscopic-radiologist rendezvous ( Fig. 4 ). After the procedure, the patient showed clinical and biochemical improvement, with resolution of the thoracic bile leak, allowing thoracic drainage to be removed. The biliary and pancreatic stents were removed 2 months later via ERCP, which also confirmed no more biliary leakage and regular emptying of contrast dye from the bile ducts after stent removal ( Fig. 5 ).

Endoscopy_UCTN_Code_TTT_1AR_2AC

